# First Complete Genome Sequences of Two Keystone Viruses from Florida

**DOI:** 10.1128/genomeA.01255-15

**Published:** 2015-10-29

**Authors:** Timothy B. Stockwell, Lea A. Heberlein-Larson, Yi Tan, Rebecca A. Halpin, Nadia Fedorova, Daniel A. Katzel, Sandra Smole, Thomas R. Unnasch, Laura D. Kramer, Suman R. Das

**Affiliations:** a J. Craig Venter Institute, Rockville, Maryland, USA; b Florida Department of Health, Bureau of Public Health Laboratories, Tampa, Florida, USA; c Department of Public Health, Massachusetts State Public Health Laboratory, Jamaica Plain, Massachusetts, USA; d Department of Global Health, Global Health Infectious Disease Research Program, College of Public Health, University of South Florida, Tampa, Florida, USA; e Wadsworth Center, New York State Department of Health, Slingerlands, New York, USA; f School of Public Health, State University of New York at Albany, Albany, New York, USA

## Abstract

We report here the first complete sequences of two Keystone virus (KEYV) genomes isolated from Florida in 2005, which include the first two publicly available complete large (L) gene sequences. The sequences of the KEYV L segments show 75.99 to 83.86% nucleotide similarity with those of other viruses in the California (CAL) serogroup of bunyaviruses.

## GENOME ANNOUNCEMENT

Keystone virus (KEYV) belongs to the California (CAL) serogroup of arboviruses in the family *Bunyaviridae* (1). The name Keystone was derived from the geographic location in Florida where it was first isolated (2). Bunyaviruses are enveloped viruses with negative-sense single-stranded RNA (ssRNA) and tripartite genomes consisting of large (L), medium (M), and small (S) RNA segments (3). The CAL serogroup contains 16 arboviruses (1) that have been associated with human cases of encephalitis (4) and influenza-like or febrile illnesses in Europe (4–6). This study generated the first two complete genome sequences for the KEYV isolated in 2005 from the Tampa Bay area, FL.

The KEYVs were amplified in Vero cells, and viral RNA was extracted from cell culture supernatant using TRIzol and chloroform. Viral cDNA was synthesized using random hexamers and oligo(dT), randomly amplified, and prepared for next-generation sequencing using a sequence-independent single-primer amplification (SISPA) method, as described previously (7). The SISPA products were normalized and pooled into a single reaction mixture that was size selected (300 to 500 bp) using the SPRIselect reagent kit (Beckman Coulter, USA) for Illumina MiSeq version 2 with 300-bp paired-end reads.

Sequencing reads were binned by bar code and trimmed to remove SISPA bar codes, random hexamer sequences, and low-quality bases, and *de novo* assembly of the reads was performed using CLC bio’s clc_novo_assemble. *De novo* contigs were searched against the GenBank nonredundant nucleotide database (NT) using BLASTN, which identified the longest contigs with high similarity matches to the KEYV M and S segments. Since no KEYV L segment sequence was available in GenBank at the time, Jamestown Canyon virus sequence was used to identify the largest *de novo* contig of the KEYV L segment in our samples. Once initial partial references were identified for each segment, these were used as scaffolds to build new reference sequences using an iterative cycle of mapping assembly employing CLC bio’s clc_ref_assemble_long program. These were then analyzed by custom software built using the J. Craig Venter Institute (JCVI)’s Jillion Framework to identify read sequences that extended beyond the reference. Multiple-sequence alignments of these sequences were then used to generate a consensus beyond the 5′ and 3′ edges of the reference. Concatenating these new consensus sequences to the previous reference created a longer sequence that was then input as a new reference in a new mapping assembly that incorporated more reads. This iterative process continued until the reference for each segment contig reached the 5′ and 3′ termini. For these genomes, all segments shared an 11-bp conserved 5′ terminal sequence, in coding sense (5′-AGTAGTGTACT-3′) and a 3′ terminal sequence, in coding sense (5′-GAGCACACTAC-3′). The final assemblies were annotated using VIGOR (8, 9) and submitted to GenBank.

Comparing these two KEYV genomes with other CAL serogroup virus genomes shows a high degree of sequence diversity; the S, M, and L segments show nucleotide similarities ranging from 67.73% to 86.15%. Although the two new KEYVs do not show a large amount of diversity (98.3% similar), the first publically available KEY L segment sequences might aid in the phylogenetic analysis of CAL serogroup viruses.

### Nucleotide sequence accession numbers. 

The full genomic sequence data are deposited in NCBI’s GenBank database under accession numbers KT630288 through KT630293.
